# Catechin-rich green tea extract increases the expression of genes of the cholesterol synthetic pathways in livers of normal diet- and high-fat diet-fed rats

**DOI:** 10.1186/2049-3002-2-S1-P71

**Published:** 2014-05-28

**Authors:** Toshikazu Suzuki, Mayumi Nagata, Ayumi Takagi

**Affiliations:** 1 Department of Health and Nutrition, Wayo Women’s University, Ichikawa, Chiba, Japan; 2 Graduate School of Human Ecology, Wayo Women’s University, Ichikawa, Chiba, Japan

## Background

*In vivo* studies using rodents have shown that green tea extract (GT) and catechins isolated from green tea can induce a variety of health effects, including anti-obesity, hypoglycemic and hypolipidemic activities. However, we and others observed that serum cholesterol levels were increased by GT and tea catechins in rats and mice [1]. In this study, we examined whether dietary GT affects the expression of genes involved in cholesterol synthesis.

## Materials and methods

Catechin-rich (30% catechin) GT was used for the *in vivo* studies. Male Sprague-Dawley rats (average weight 237.7 g) were divided into six groups. The first group was fed a normal diet (ND, 10% calories from fat); the second group a high-fat diet (HFD, 40% calories from fat); the third group a ND containing 1% GT (ND + 1% GT); the fourth group a HFD + 1% GT; the fifth group a ND + 3% GT; and the sixth group a HFD + 3% GT. After 4 weeks of feeding, rats were euthanized by whole blood collection under anesthesia. Serum metabolites were determined using a dry chemistry analyzer. Total RNA samples extracted from the liver were used for microarray and quantitative real-time PCR (qPCR) analyses. Hepatic lipids were extracted with chloroform-methanol 2:1, and liver triglyceride (TG) and cholesterol (Cho) levels were determined.

## Results

As reported previously, GT increased the serum levels of total cholesterol (T-Cho) and high-density lipoprotein cholesterol (HDL-Cho) in a concentration-dependent manner in both ND- and HFD-fed rats. Conversely, GT lowered hepatic TG and T-cho levels. Microarray analysis data suggested that GT increased more than two-fold the mRNA expression of nine hepatic genes involved in cholesterol synthesis out of 21 analyzed in ND-fed rats. qPCR analysis revealed that GT significantly increased the mRNA levels of six genes involved in cholesterol synthesis in ND-fed rats. Similar but smaller effects of GT also were observed in HFD-fed rats.

## Conclusions

The results suggested that GT increased serum cholesterol levels, at least in part, through the increased expression of genes in the cholesterol synthetic pathways in the liver. GT also increased the mRNA levels of *Srebf2* and *Insig1*, which are positively regulated by SREBP2. Therefore, dietary GT may increase expression of these genes by SREBP2 activation.
